# Red and blue states: dichotomized maps mislead and reduce perceived voting influence

**DOI:** 10.1186/s41235-023-00465-2

**Published:** 2023-02-09

**Authors:** Rémy A. Furrer, Karen Schloss, Gary Lupyan, Paula M. Niedenthal, Adrienne Wood

**Affiliations:** 1 Harvard Medical School, Boston, USA; 2 University of Virginia, Charlottesville, USA; 3 University of Wisconsin – Madison, Madison, USA

## Abstract

In the United States the color red has come to represent the Republican party, and blue the Democratic party, in maps of voting patterns. Here we test the hypothesis that voting maps dichotomized into red and blue states leads people to overestimate political polarization compared to maps in which states are represented with continuous gradations of color. We also tested whether any polarizing effect is due to partisan semantic associations with red and blue, or if alternative hues produce similar effects. In Study 1, participants estimated the hypothetical voting patterns of eight swing states on maps with dichotomous or continuous red/blue or orange/green color schemes. A continuous gradient mitigated the polarizing effects of red/blue maps on voting predictions. We also found that a novel hue pair, green/orange, decreased perceived polarization. Whether this effect was due to the novelty of the hues or the fact that the hues were not explicitly labeled “Democrat” and “Republican” was unclear. In Study 2, we explicitly assigned green/orange hues to the two parties. Participants viewed electoral maps depicting results from the 2020 presidential election and estimated the voting margins for a subset of states. We replicated the finding that continuous red/blue gradient reduced perceived polarization, but the novel hues did not reduce perceived polarization. Participants also expected their hypothetical vote to matter more when viewing maps with continuous color gradations. We conclude that the dichotomization of electoral maps (not the particular hues) increases perceived voting polarization and reduces a voter’s expected influence on election outcomes.

**Supplementary Information:**

The online version contains supplementary material available at 10.1186/s41235-023-00465-2.

## Significance statement

The media uses electoral maps to visualize the results of political elections. In the United States, red represents the Republican party and blue represents the Democratic party in a winner-takes-all election. This dichotomization provides a simple visual representation of election outcomes, but it conceals the margin of votes by which an election is won/lost. The present experiments distinguish the perceptual influence of categorical representation from the effect of conditioned color associations on perceived voting polarization. Dichotomous color mappings increase perceptions of polarization and decrease expectations of individual voters’ influence compared to continuous hue-lightness gradients, regardless of whether the hues are red/blue or a novel hue pairing. These findings offer practical implications for data visualization in electoral maps. Switching from dichotomous coloring to continuous gradations can help mitigate the polarizing effects of red/blue maps on voting estimates and increase perceived voting influence.

## Introduction

In current United States politics, the association between *Democrats* and *blue*, and *Republicans* and *red*, is so ubiquitous that it is easy to forget how recently this color mapping emerged. A person flipping through TV channels during a pre-2000 election season might have seen, on one news station, a political forecast map coded Republican-leaning districts or states as blue and Democrat-leaning ones as red, but then the next news station might use red–blue, or orange–green, or no hues at all (Bensen, 2003; Farhi, 2004; Enda, 2012). There was no agreement across news outlets or across election seasons, and if anything, left-leaning political parties had a stronger association with red (e.g., the Communist Party). The Republican-red and Democrat-blue mapping took hold in 2000, as the Bush v. Gore presidential election controversy and vote recounts unfolded over days and weeks and received extensive news coverage. Media outlets covering the election gradually settled on the current party-color mapping, and the associations stuck.

In just two decades a color-coding system that began as a convenient visualization tool has become a symbol for—and, potentially, a contributor to—the increasing divide between liberals and conservatives. The symbolic dichotomization implies a lack of nuance in political ideology, which in turn might exacerbate the bipartisan divide across political (Baldassarri & Gelman, 2008), geographic (Tam Cho et al., 2013), and virtual (Bail et al., 2018) settings. The existence of “red states” and “blue states” is now taken for granted, though in actuality the U.S. is a quilt of different shades of purple. This was demonstrated by Robert Vandervei, who coined the phrase *Purple America* after the 2000 presidential elections (Klinkner, 2004). Red and blue have become such essential properties of people’s concepts for *Republican* and *Democrat* that on election days, when the parties are made particularly salient, people’s personal color preferences become more aligned with their party affiliation (Schloss & Palmer, 2014). That is, people’s preferences for their political in-group permeates their color preferences, underscoring the strength of the association between parties and colors.

**Fig. 1:**
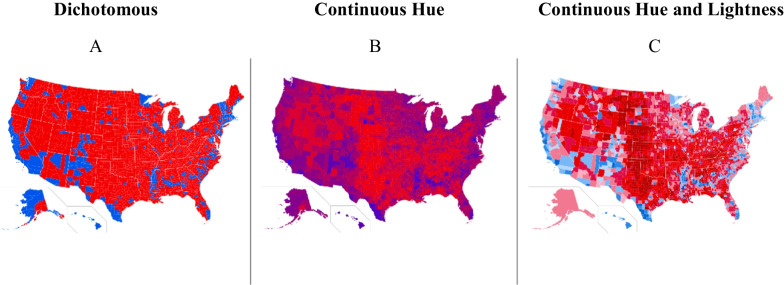
Three different approaches to designing red/blue maps that represent the outcome of the 2016 election at the county level. **A:** Dichotomous solution. The counties where Donald Trump received the most votes are colored red and the counties where Hillary Clinton received the most votes are colored blue. **B:** A continuous hue solution. Counties are shaded according to winning candidate’s percentage of the vote, with purple counties being closer races and redder or bluer counties having a larger margin in favor of Trump or Clinton, respectively. **C:** A continuous hue and lightness solution, where base hue (red or blue) indicates which candidate won the county, and lightness indicates the margin by which a candidate won a county (darker colors indicate larger margin). Prior work suggests judgments about polarization are reduced by solution B relative to solution A, but no work has compared solution C (Rutchick et al., 2009). Furthermore, no work has compared the effect of solution A relative to an absence of red–blue coding or to an arbitrary hue mapping to isolate general effects of hue on category representation from the effect of prior knowledge about the significance of red and blue in politics

Depicting the U.S. using a dichotomous color mapping simplifies complex voting patterns (see Fig. 1A), but it can also alter the ways in which people construe partisan politics, potentially further polarizing people’s estimates of voting outcomes. Color facilitates categorization (Oliva & Schyns, 1996) and grouping of objects (Goldstone & Hendrickson, 2010; Makovski & Jiang, 2009; Wertheimer, 1923), so people can look at a color-coded map and quickly extract information. But maps utilizing the red–blue categorization have a significant drawback. Prior work shows that dichotomizing a political map using two hues, red and blue, rather than a continuous hue gradient (from red to blue, through purple), leads people to overestimate within-state political homogeneity and nation-wide political polarization (Rutchick et al., 2009). In other words, people assume “blue states” must be substantially more Democratic and liberal than “red states”, when in fact they could be just a few percentage points apart. This exaggeration occurs because a categorical classification scale is applied to a continuous feature (e.g., the percent of voters in a geographic region that hold a particular opinion), which causes people to hold more categorical representations of the feature (Szafir et al., 2016). People may then represent residents of “red states” and “blue states” as more different from each other than they actually are, potentially exacerbating harmful stereotypes about “liberal bubble states” and conservative “flyover country.”

As depicted in Fig. 1, there are multiple ways of using continuous gradations of color to represent a variable of interest (e.g., the percent of votes that went to a given candidate, or the percent of voters who endorse a position). Sticking with the red–blue representation, such a map could use a hue gradient that passes from pure red, through purple (the result of combining red and blue), to pure blue (Rutchick et al., 2009). This would result in a map of various hues that capture the continuous distribution of voting patterns, as discussed in the previous paragraph (see Fig. 1B). However, these purple maps make it challenging to interpret the outcome of a winner-takes-all electoral college system, where a single percentage point can decide whether an entire state goes to the Republican or Democratic candidate. For such an election system, it is useful to represent the actual or likely *winner* of a district or state, as well as the *margin* by which they won or are expected to win. One way to maintain the continuous representation supported by red–purple–blue maps, while illustrating the categorical outcome of a winner-take-all system, is to use a divergent map with two hues interpolated through an achromatic color, like white (see Fig. 1C). This visualization method avoids the muddiness of purple shades, but it is yet unknown whether they eliminate the categorical effects of hue on people’s judgments of polarization (Jacobson, 2016).

In two studies, we asked whether dichotomizing maps into red and blue states leads people to overestimate polarization compared to maps that represent voting patterns using a continuous gradation from red to white to blue. We also investigated the effects of prior color-concept associations by testing whether maps designed with novel, non-party hues (orange/green) decrease perceived voting polarization compared to the traditionally conditioned associations of red/blue hues with US political parties.

We tested three hypotheses across two pre-registered studies. The first hypothesis was that hues associated with political parties (red/blue) increase perceived voting polarization compared to previously non-associated hues (orange/green) or uniform grey. The second hypothesis was that dichotomous color maps increase perceived voting polarization compared to continuous color maps. The third hypothesis was that the effect of dichotomous and continuous color maps depends on whether the hues had prior political associations (i.e., an interaction effect). The two experiments tested the same three hypotheses, under two different contexts. In Study 1 participant predicted future voting patterns of swing states for which there were thus no clear base rates on which they could base their responses. In Study 2 participants recalled results from the 2020 presidential election for a random subset of all 50 states to determine whether the effects only applied to swing states. Study 2 also tested a possible consequence of perceiving red and blue states to be highly polarized: thinking that a single vote in those states will matter less.

## Study 1

### Methods

We preregistered the study design, target sample size, and exclusion criteria (https://osf.io/tk7rq). Any deviations from the preregistered plan are explicitly noted. This study was approved by the Institutional Review Board at the University of Virginia.

#### Participants

On October 30, 2020—four days before the 2020 presidential election—we recruited 550 participants via Amazon’s Mechanical Turk to participate in a 10-min study about political judgments in exchange for $1.50 ($9/hr). We recruited participants who were 18 years or older, were American citizens, had lived in the United States for at least 10 years, and self-identified as being fluent in English.

The preregistered sample size (500) was determined with a power analysis. Prior work examining the effect of color coding on political judgments, using a task similar to the Polarization Task of the present study (Rutchick et al., 2009), found an effect size estimate of η^2^_p_ = 0.03. Based on this prior effect size, a power analysis using the modelPower() function from the lmSupport R package suggested 500 participants would give us 97.5% power to detect the Polarization Task effect (with alpha = 0.05 and η^2^_p_ = 0.03).

We over-recruited by 50 participants to ensure we would have sufficient power even after excluding participants for failing our bot and attention checks. Initially 991 people started the survey, but 387 did not reach the consent form because they failed the initial bot check. Of the remaining 604, 99 failed to pass the mid-way attention check, leaving data from 505 participants for analysis. We had planned to eliminate data from any participants who left their browser during the tasks or reported color vision deficit, but we failed to record these pieces of information. Additionally, we decided not to eliminate participants who failed a final manipulation check because it was too stringent: in response to an open-ended debriefing question asking them what they thought the study was about, we planned to only keep data for participants who mentioned a relevant word stem (map*, politic*, color*, vot*, Democrat*, Republican*, u.s*, us*, or united states*). Within the 505 participants included, this criterion would have reduced our sample to 261. We did not anticipate that participants would use other words and tell us how they feel about the study instead.

The final sample of 505 participants included 173 women, 305 men (1 transgender man), and 1 who identified as neither woman nor man. Participants had a median age bracket of 35–44 years (16 were ages 18–24, 215 were ages 25–34, 143 were ages 35–44, 60 were ages 45–54, 23 were ages 55–59, and 23 were ages 60+). Participants reported their ethnicities as follows: 362 White, 61 Black/African American, 29 Asian/Pacific Islander, 12 Hispanic/Latino/a, 12 Native American, and 4 “other”. 54 of the participants had a high school degree, 45 had a 2-year college degree, 279 had a 4-year college degree, 93 had a Master’s degree, and 9 had a PhD or professional degree.

In terms of the political leanings of the participants, 196 were registered Democrats, 192 registered Republicans, 43 Independent/Other, 39 unaffiliated, 10 not registered and 25 missing responses. 214 participants were voting for Biden, 235 for Trump, 19 were not voting and there were 37 missing responses.

#### Design

Materials for the study can be found on OSF (https://osf.io/gq8f2/). These include the informed consent sheet, a PDF showing the sample tasks and colormap stimuli sets for both studies.

Participants first read a consent information page inviting them to “participate in a research study about people's understanding of political attitudes” and telling them “The purpose of the research is to gain a better understanding of how people think about American politics and the current political climate. This study will include a random sample of voting-age Americans.” Participants were then randomly assigned to one of five between-subject conditions: dichotomous red/blue, dichotomous orange/green, continuous red/blue, continuous orange/green, and uniform grey. After completing the task, they answered questions about their knowledge of the 8 states included in the current study, their political opinions and activity level, and basic demographic information. We then debriefed them, thanked them for their time, and gave them their MTurk payment codes.

**Fig. 2:**
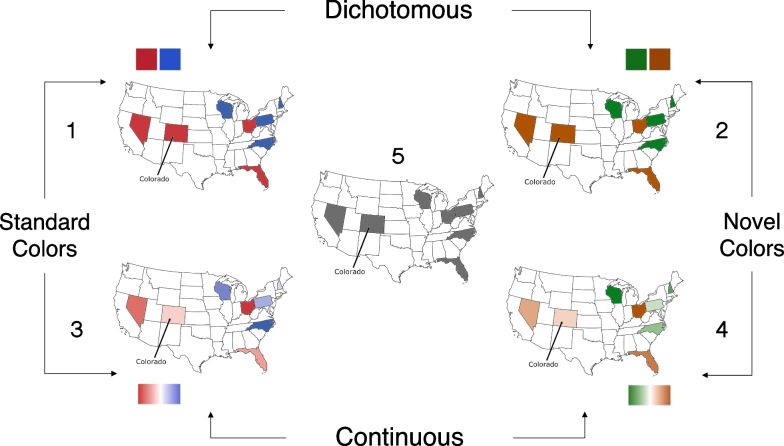
Sample Polarization Task stimuli for each of the five between-subject conditions: (1) red/blue dichotomous, (2) red/blue continuous, (3) orange/green dichotomous, and (4) orange/green continuous, and (5) uniform gray. Within the four hue varying conditions, the colors assigned to each state were randomized across participants, but the states were always divided evenly across the two hue categories. On each of the eight trials a participant completed, the position of the line with the arrow identified which of the 8 states the participant was judging. Note that the colors assigned to each state remained constant across a single participant’s trials

Participants estimated the likely presidential voting pattern of 8 states for a “hypothetical” Republican or Democratic candidate. The states were chosen because they have been classified as “swing states” in recent elections (Silver, 2016). The task involved a 2 × 2 between-subjects design: 2 Hue Pairs (red/blue versus orange/green) × 2 Gradient Steps (dichotomous versus continuous). A fifth condition with uniform gray maps was added to the 2 × 2 design and served as a baseline. The maps, as depicted in Fig. 2, were colored using either (a) dichotomized red/blue, (b) dichotomized orange/green (to determine whether the dichotomizing effects of red/blue maps are entirely due to prior associations people have between those hues and political parties), (c) continuous red/green passing through white, (d) continuous orange/green passing through white, or (e) uniform grey (to establish a baseline for how polarized participants judge the 8 states to be). We elaborate on the color specification details below.

**Fig. 3:**
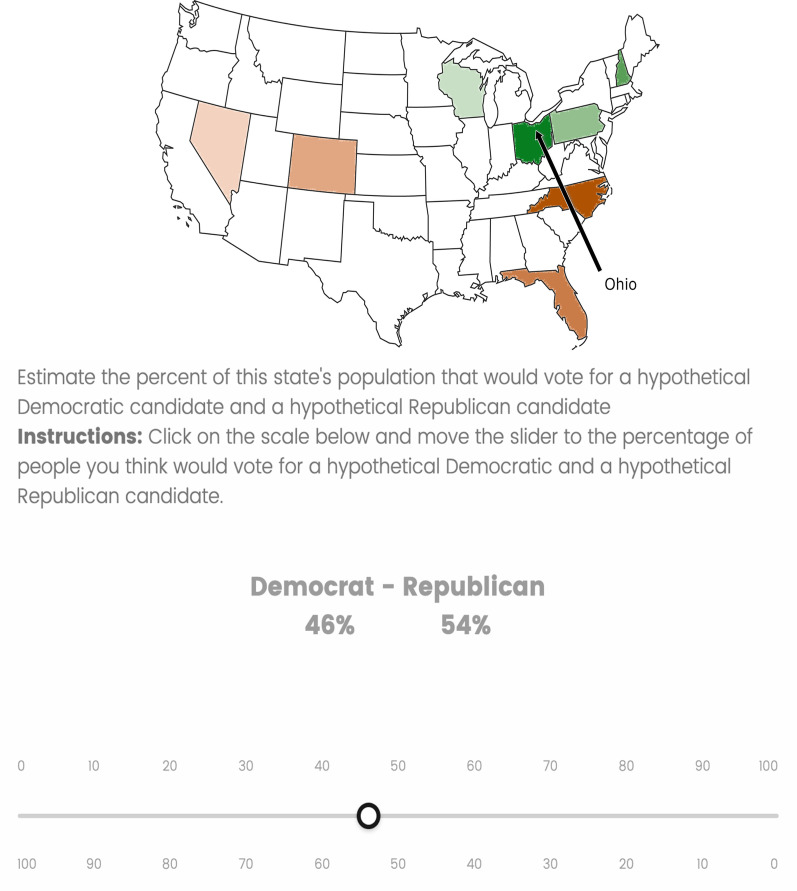
Sample Polarization Task trial. Participants used a slider scale which interactively depicted the predicted voting estimates of both Democrat and Republican parties as they moved the cursor

Polarization was measured by having participants make voting predictions ranging from 0 to 100% of the state’s voting percentages for a Democrat versus Republican candidate (see Fig. 3). Higher polarization is quantified as greater absolute difference in the predicted voting breakdown (percent Democrat versus Republican) for states of different hues. For instance, predicting blue states will vote 60% Democrat and 40% Republican, and red states will vote 40% Democrat and 60% Republican, constitutes a more polarized voting estimate than predicting all states will go 50% Democrat and 50% Republican.

The task consisted of 8 randomized trials (one trial for each of the 8 swing states) in which participants were asked to predict what percent of the state’s voting population would vote Republican versus Democratic in an election with hypothetical candidates.

##### Map stimuli

We chose 8 U.S. states identified as “swing states” by FiveThirtyEight, a political and data analysis blog site that specializes in polling predictions based on analysis of big data (Silver, 2016). Swing states are those which either a Republican or a Democratic candidate has a reasonable chance of winning and which have been closely contested in recent elections. The FiveThirtyEight analysis identified 12 states as “swing states”: Colorado, Florida, Iowa, Michigan, Minnesota, Ohio, Nevada, New Hampshire, North Carolina, Pennsylvania, Virginia, and Wisconsin. We selected eight states from this list that were geographically dispersed and, as much as possible, non-contiguous: Colorado, Florida, Ohio, Nevada, New Hampshire, North Carolina, Pennsylvania, and Wisconsin.

The map was a vector drawing of the state borders of the continental United States with black lines on a white background. The dichotomous map included pairs of “base colors” (red/blue, orange/green), which varied in hue but had the same lightness (L*) and chroma (C*) in CIEL Ch space. The colors initially were defined in CIELCh space, converted to CIELAB space, and then converted to RGB values using standard assumptions in the MATLAB lab2rgb function (see Table 1). Given that participants viewed the colors on their own uncalibrated devices, the exact colors observed varied somewhat across participants, which mimics variations in viewing conditions when participants look at election results on their own devices.

**Table 1:**
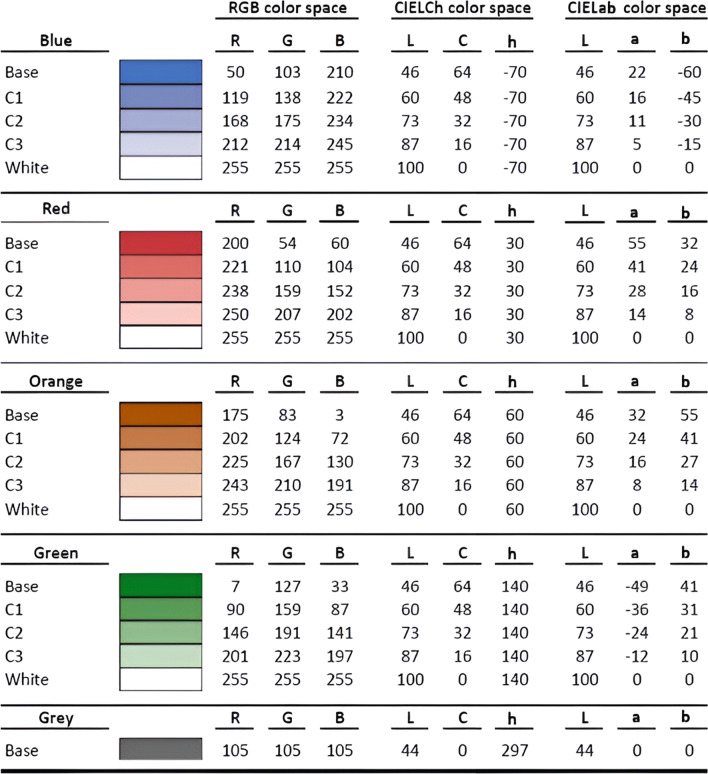
Color coordinates for all test colors, specified in RGB, CIE LCh and CIELAB color spaces

Within each hue, there is a base color (blue, red, orange, green), plus three colors interpolated between the base color and white (labeled C1–C3) to produce a color scale. Note that the "Orange" colors may appear brown, but we use the term "Orange" to refer to the hue (dark orange can appear brown)

We generated the dichotomous red–blue and orange–green maps by filling four of the swing states with one hue in the pair and four swing states with the other hue in the pair (see maps 1 and 2 depicted in Fig. 2 and the color coordinates in Table 1). We balanced the hue in which the 8 states were displayed across participants by generating maps for all possible hue-state permutations (requiring four states per hue). This resulted in 70 dichotomous red–blue and 70 dichotomous orange-green maps.

We generated the continuous maps by first defining a 4-step “color scale” for each base color, which included the base color and three steps interpolated between the base color and white (interpolation computed in CIELAB space with white set to L* = 100, a* = 0, b* = 0). The gradation between the base color and white varied in lightness and chroma, but we will subsequently refer to the steps in terms of lightness for simplicity. Starting with the dichotomized maps described above, we filled the four states within each hue using the 4-step color scale, one color per state (see right panels, Fig. 2). For instance, in a continuous red–blue map, one of the four states assigned to be red was filled with the base red and the other three with increasingly lighter reds (for color coordinates, see Table 1). We generated 70 maps for the red–blue gradient condition and 70 for orange-green gradient condition by randomly assigning one of the 8 colors to each of the 8 states. Note that we did not generate all possible permutations of lightness assignment because that would require generating 8!, or 40,320, maps. We generated the uniform gray map by filling all of the eight swing states with gray. The gray in the uniform gray map had the same lightness as the base colors, but chroma was set to zero.

##### Procedure

Prior to providing informed consent and starting the actual study, participants completed a botcheck that required them to click on three circles in order to match the colors indicated in the instructions. Only 61% (604 out of 991) of participants passed the botcheck and were permitted to start the study, as preregistered. Before the task began, participants were told, “DO NOT refer to any sources (such as Google) while doing this task. We are interested in your general impressions of politics in the United States, not in your knowledge or accuracy.” On each trial, participants saw a vector line drawing of a political map of the continental United States with an arrow pointing to one of the eight swing states (see Fig. 3). Below the map was a bimodal slider scale (Democrat–Republican) which displayed cumulative percentages for each political party with the prompt, “Estimate the percent of this state's population that would vote for a hypothetical Democratic candidate and a hypothetical Republican candidate.”

Importantly, the instructions did not explain the meaning or relevance of the color of the states, leaving participants to draw their own inferences about the color’s meaning. Participants had unlimited time to make a response on each of the eight trials (one per state), which were presented in random order. Following completion of the Polarization Task, participants saw an attention check that simply asked them to click the state of California on a labeled map. Of the 604 participants who made it to this point, 505 (84%) passed the attention check—the remaining 99 were excluded from analyses, as preregistered.

##### End of study questionnaire

In the final phase, participants indicated which of the two political parties from the 2016 election won the majority of votes in each of the states from the Polarization Task. Participants also reported how familiar they were with each state on a 5-point Likert scale ranging from 1 (not familiar at all) to 5 (extremely familiar). These questions were included for possible follow-up exploratory analyses.

Once participants’ prior knowledge about the 8 swing states was assessed, they reported their political attitudes and perceptions of political polarization. The following items were included as potential moderators in exploratory analyses. Political attitudes were measured using two items: “How do you feel about the Democratic party in general?” and “How do you feel about the Republican party in general?” ranging from “Dislike a great deal” to “Like a great deal” on a 100-point slider scale. Perceived polarization was measured on a 7-point Likert scale ranging from strongly agree to strongly disagree using four items: “Do you think the United States is more politically divided now than in the past?”; “Do you think political polarization is a problem in our country?”; “Do you think half of the country is being ignored by politicians?”; and “Do you think Red state Americans and Blue state Americans ultimately share the same values?” Participants were then asked the following open-ended questions: “What do you think this study is about?”, “Did the colors on the maps make you think of anything?”, and “Which colors did you see on the maps? Did you associate them with a particular political party?” They then reported who they voted for in the 2016 presidential election and who they intend on voting for in the 2020 election. Finally, participants completed a standard demographic questionnaire.

## Results

All analyses were conducted using R. Deviations from the preregistered analysis plan (https://osf.io/tk7rq) are noted below. See https://github.com/adriennewood/red-state-blue-state for both planned and final analysis syntax, output from analyses conducted on both simulated data and the real data. The data are also openly available (https://osf.io/tk7rq).

### Estimating polarization

Participants were randomly assigned to a color condition: dichotomous red–blue dichotomous orange-green, continuous red–blue, continuous orange–green, and uniform grey. Each participant completed 8 trials, judging one state on each trial. We calculated how “polarized” a participant’s voting predictions were by calculating the relative variability in their responses for all the states depicted with the same hue versus the same states randomly depicted with the alternative hue. In other words, we conducted an ANOVA separately for each participant (Ntrials = 8), and used the resulting F statistic, which indicated how distinct their judgments were for each map color, as our measure of polarization (see Additional file 1 for a visual explanation). For the uniform grey condition, we randomly labeled each response as belonging to “color 1” or “color 2.” This allowed us to treat participants’ polarization scores in the uniform grey condition as baseline polarization scores that might be achieved by chance. We did not preregister this final step and originally planned to simply exclude the uniform grey participants from certain analyses.

We calculated secondary polarization scores with the aim of distinguishing between two different types of potential polarization: spread and clustering. The F statistic, our primary measure of polarization derived from an ANOVA, is calculated by dividing the Mean Squares Between (MSB) by the Mean Squares Within (MSW). MSB is calculated by dividing the Sum of Squares Between (SSB) by the Degrees of Freedom Between, while MSW is calculated by dividing the Sum of Squares Within (SSW) by the Degrees of Freedom Within. Polarization, as measured by calculating an F statistic for each participant based on their responses across the 8 trails, can be influenced by both spread (measured by SSB) and clustering (SSW), which are two different features of polarization. The SSB for a participant captures the average variation of their voting margin predictions between states that are randomly assigned to different colors. The SSW for a participant captures the average variation for which a participant predicts states of the same color to vote.

Based on simulated polarization data, we expected all three measures (F statistic, SSB, and SSW) to be positively skewed. We preregistered the application of a log transformation in this case, which sufficiently corrected the skew of the SSB and SSW scores. However, the log-transformed F statistic polarization scores were still skewed, so we raised them to the ¼ power (F^1/4^).

### Preregistered analyses of the effect of hue and gradation on predicted voting polarization

In a series of mixed effects models, we regressed the polarization estimates (transformed participant-level F, SSB, and SSW scores) on contrast-coded variables for Hue Pair (red/blue = RB, orange/green = OG, and uniformly grey = UG) and Gradient Steps (dichotomous = D and continuous = C). In the following analyses we use Hue Pair to refer to contrasts including the two hue pairs (red/blue and orange/green) as well as (achromatic) grey.

#### Model 1: The effect of Hue Pair on predicted voting polarization from dichotomous maps

The first linear regressions tested hypothesis 1 by comparing participants in the dichotomous (Orange/Green and Red/Blue) or uniform grey conditions. We predicted that within the dichotomized map conditions, participants would make more polarized voting predictions for maps with strong party hue associations (red/blue) than for the map without strong party hue associations (orange/green). We also hypothesized that participants would make more polarized voting predictions for both dichotomous maps compared to a uniform grey map.

We regressed participant’s polarization scores on two orthogonal contrast codes comparing the three-color conditions (red/blue, orange/green, grey). The linear contrast variable (Uniform Grey = − 0.5, Dichotomous Orange/Green = 0, Dichotomous Red/Blue = 0.5) tested the hypothesized increase in polarization from Uniform-Grey, to Orange/Green, to Red/Blue. The quadratic contrast variable (Uniform Grey = − 1/3, Dichotomous Orange Green = 2/3, Dichotomous Red/Blue = − 1/3) tested the hypothesis that the Orange-Green polarization scores were different from the Uniform-Grey and Red–Blue.

##### Polarization

**Fig. 4:**
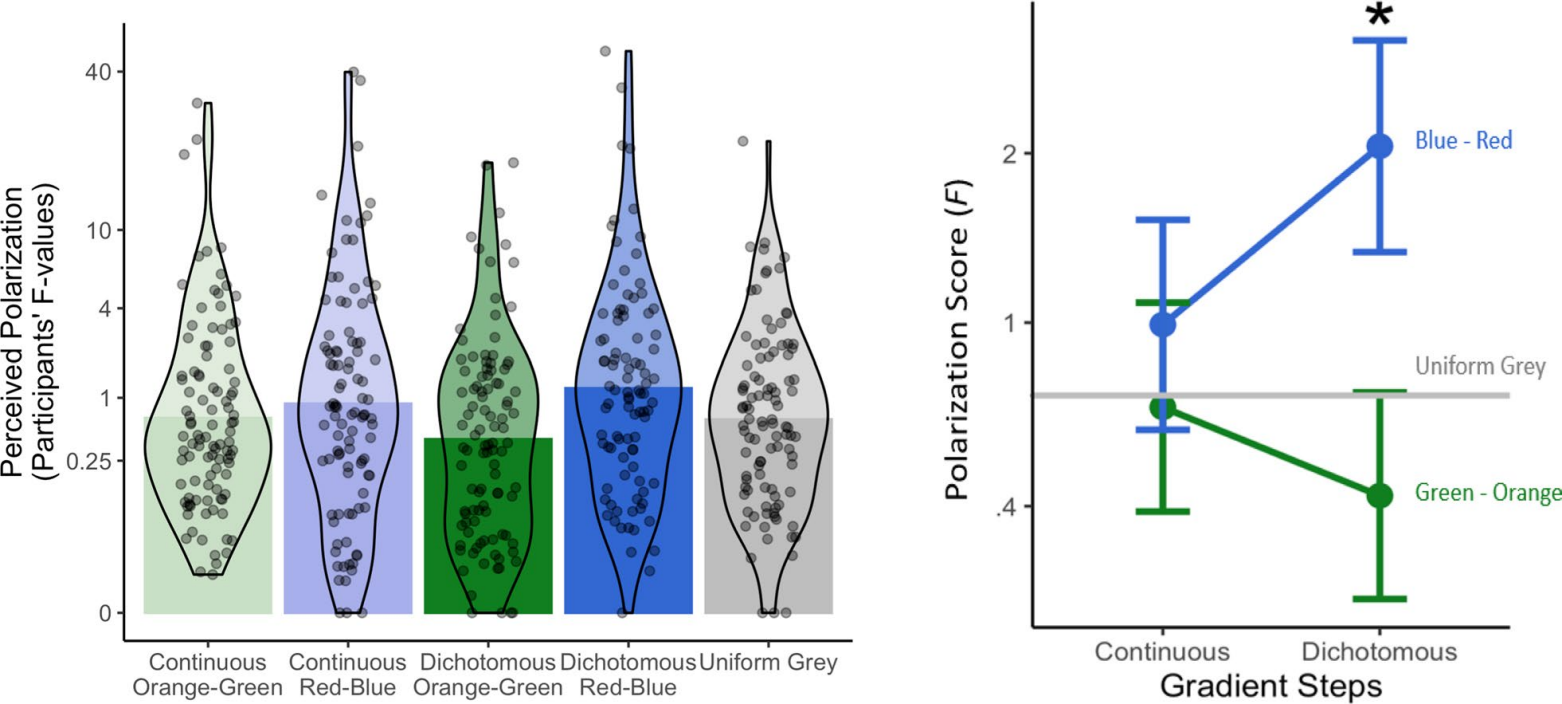
Polarization of judgments (participant-level F-values) in the Polarization Task across the five between-subject conditions are presented on the left. Estimates from Model 2 are presented on the right. The interaction between Gradient Steps and Hue Pair was significant, as was the simple effect of Hue Pair for Dichotomous maps. Note that the F-values depicted in these plots are untransformed for ease of interpretability but were raised to the ¼ power for the analyses to correct for positive skew

As hypothesized, the linear contrast for participants’ judgment polarization (participant-level F-values^1/4^) was significant, *b* = 0.278, *SE* = 0.085, *t*(300) = 3.26, *p* = 0.001, η_p_^2^ = 0.034. However, the orthogonal contrast was also significant, *b* = − 0.251, *SE* = 0.073, *t*(300) = − 3.43, *p* < 0.001, η_p_^2^ = 0.038 showing that participants made the most polarized voting predictions when viewing the dichotomous red–blue maps (untransformed F-values, *M* = 17.80, *SD* = 69.19) compared to the grey (*M* = 2.34, *SD* = 7.90) and dichotomous orange-green maps (*M* = 1.55, *SD* = 3.24), but the grey maps unexpectedly appeared in between the red–blue and orange-green in terms of polarization (see Fig. 4). A regression comparing the grey condition to the orange/green condition (combining continuous and dichotomous conditions) revealed that they were not significantly different from each other, *b* = − 0.063, SE = 0.055 *t*(304) = − 1.146, *p* = 0.253, η_p_^2^ = 0.004.

##### Cluster and spread

The participant-level SSW scores—how tightly clustered participants’ voting predictions were within-hue—were not significantly predicted by the two contrast variables, |*t*’s|< 0.95, *p*’s > 0.34. The overall effect of hue on F-values thus seemed to be carried by differences in how spread out (SSB) participants' voting predictions were between hue-level state groupings: linear contrast code, *b* = 0.521, *SE* = 0.290, *t*(300) = 1.79, *p* = 0.074, η_p_^2^ = 0.012; orthogonal quadratic contrast code, *b* = − 0.839, *SE* = 0.249, *t*(300) = − 3.37, *p* < 0.001, η_p_^2^ = 0.037.

#### Model 2: The interaction between Hue Pair and Gradient Steps on predicted voting polarization

The second set of linear regressions tested hypotheses 2 and 3 by comparing the dichotomous and continuous maps. Hypothesis 2 held that polarization in voting predictions would be greater for dichotomous maps than continuous maps for both hue pairs (red/blue and orange/green). Hypothesis 3 predicted there would be an interaction between party association and dichotomization for perceived voting polarization—the effect of Hue Pair (red/blue versus orange/green) on polarization in voting estimates would be greater for dichotomized compared to continuous maps. In other words, depicting political parties with non-traditional hues (orange/green), in addition to using alternate (continuous) gradient steps may interact to further reduce predicted polarization.

We regressed polarization—and, secondarily, SSW and SSB—on Hue Pair (orange/green = -0.5, red/blue = 0.5), Gradient Step (continuous = -0.5, dichotomous = 0.5), and their interaction. The Gradient Step contrast tested hypothesis 2, that polarization would be greater for dichotomous than continuous maps. The interaction term tests hypothesis 3, that the effect of Hue Pair would be dampened when the gradient was continuous rather than dichotomous.

##### Polarization

The effect of Hue Pair was again significant when the continuous maps were included, with more polarized predictions for red/blue maps than the orange/green maps, *b* = 0.241, *SE* = 0.059, *t*(398) = 4.06, *p* < 0.001, η_p_^2^ = 0.040. The main effect of Gradient Steps was not significant, *p* = 0.340, so hypothesis 2 was not supported. The interaction between Hue Pair and Gradient Steps was significant, however, supporting hypothesis 3, *b* = 0.298, *SE* = 0.119, *t*(398) = 2.51, *p* = 0.013, η_p_^2^ = 0.016. The interaction is apparent in Fig. 4 (left).

To interpret the interaction between Hue Pair and Gradient Steps, we re-centered the Gradient Steps variable and reran the model to estimate the simple effects of Hue Pair when the maps were dichotomous and when they were continuous (note: we did not preregister this test of the simple effects). The continuous red/blue maps (untransformed F-values, *M* = 4.45, *SD* = 15.84) were significantly less polarizing than dichotomous red–blue maps (*M* = 17.80, *SD* = 69.19; *b* = 0.199, *SE* = 0.085, *t*(398) = 2.36, *p* = 0.019, η_p_^2^ = 0.014). However, there was not a significant difference between continuous orange/green maps (*M* = 1.99, *SD* = 4.54) and dichotomous orange/green maps (*M* = 1.55, *SD* = 3.24;* p* = 0.237). These results suggest that dichotomization only polarizes voting predictions when the hue pairs have prior associations with the groups they represent.

##### Cluster and spread

Neither Hue Pair, Gradient Steps, nor their interaction significantly affected how clustered participants’ predictions were within-hue (SSW), |*t*’s|< 1.5, *p*’s > 0.14. The pattern for spread (SSB), or the difference in participants’ judgments between hue types, again mirrored the pattern for polarization (F values). The main effect of hue was significant, *b* = 0.593, *SE* = 0.207, *t*(399) = 2.97, *p* = 0.004, η_p_^2^ = 0.020, with greater between-hue variance for red/blue maps compared to orange/green maps. The main effect of Gradient Steps was not significant, *p* = 0.566, but the interaction between Hue Pair and Gradient Steps was significant, *b* = 1.014, *SE* = 0.414, *t*(399) = 2.45, *p* = 0.015, η_p_^2^ = 0.015, demonstrating that continuous maps decreased predicted voting polarization compared to dichotomous maps, only when there were salient associations (blue-Democratic, red-Republican).

### Study 1 summary of findings

Hypothesis 1 stated that red/blue voting predictions would be more polarized than orange/green predictions, followed by grey predictions. The central hypothesis—that red/blue maps would result in the most polarized voting predictions—was supported. However, the voting predictions for grey maps were unexpectedly more polarized than the predictions for orange/green maps. Hypothesis 2 stated that the dichotomous gradient steps (for both red/blue and orange/green maps) would lead to more polarized voting predictions than the  continuous hue and lightness gradients. This hypothesis was supported for red/blue maps, but, unexpectedly, was not supported for orange/green maps. Hypothesis 3, which predicted an interaction between Gradient Steps and Hue Pair, was supported.

## Discussion

As hypothesized, participants’ voting predictions were more polarized when maps used party-associated hues (red/blue) compared to arbitrary hues (orange/green). We further found that when people had salient associations between hues and groups (red-Republican and blue-Democratic), continuous maps decreased predicted voting polarization compared to dichotomous maps. When there were no prior conditioned associations (green-Democratic, orange-Republican) dichotomization had no significant effect on predicted voting polarization. Finally, polarization was lessened for uniform grey maps and orange/green maps compared to dichotomous red/blue maps.

In Study 1, the polarizing effects we observed were driven by the spread (predicted difference between two states of different hues), rather than the clustering (predicted similarity between two states of the same hue). Thus, changing the hue and gradient steps of a political map appear to have greater impact on the predicted between-group difference than on the predicted within-group consistency. We did not find moderating effects of conditions based on political ideology (measured by intended voting choice for the 2020 presidential election) or general concerns about polarization. While people may be concerned with general bipartisan polarization, such a concern does not influence their predictions about voting patterns.

The present study design has several limitations, which may hinder the generalizability of these findings. (1) We only used swing states and instructed participants to imagine hypothetical candidates in order to limit the influence of participants’ prior knowledge, but we cannot know whether the results generalize beyond states that historically have close voting margins. (2) We let participants freely infer the meaning of the hue pairs used in electoral maps (red/blue and orange/green) to see whether the red-Republican and blue-Democrat association occurs implicitly in contrast to novel hue pairs, but it is therefore not clear whether the novel hue pairs (green/orange) decreased perceived polarization because of their lack of association, or whether they were ignored and participants, due to the uncertainty, selected the mid-point of the scale. (3) We asked participants to estimate voting patterns in a hypothetical future election, so we had no true baseline against which to compare their estimates.

We designed a second study with the aim of increasing the ecological validity and generalizability of these findings. Study 2 used electoral maps showing actual results from the 2020 Presidential Election and asked participants to estimate the voting margins. Participants were again assigned to view maps showing one of four hue-gradient combinations. Each participant evaluated a subset of 15 States (out of 50 US States). Finally, we explored the influence of hue pairs and gradients on a secondary dependent variable: how much participants thought a single vote matters in each state.

## Study 2

### Methods

We preregistered the study design, target sample size, and exclusion criteria (https://aspredicted.org/8S6_95S). This study was approved by the Institutional Review Board at the University of Virginia.

#### Participants

On July 1, 2022, we aimed to recruit 800 mTurk participants via Cloud Research to participate in a 10-min study about political judgments in exchange for $1.50 ($9/hr). We recruited participants who were American and 18 years or older.

Of the 905 participant observations recorded, 851 participants reached the main task. Of those participants, 53 failed to identify California on a map and were removed and 18 said they received outside help, leaving a total sample size of 780 participants.^1^

The final sample included 393 women, 380 men, 4 who identified as transgender and 3 who did not identify with these options). Participants’ age ranges were the following: 47 were ages 18–24, 262 were ages 25–34, 223 were ages 35–44, 139 were ages 45–54, 38 were ages 55–59, and 71 were ages 60+). Participants reported their ethnicities as follows: 577 White, 83 Black/African American, 50 Asian/Pacific Islander, 46 Hispanic/Latino/a, 5 Native American, and 19 “other.” 183 of the participants had a high school degree, 105 had a 2-year college degree, 342 had a 4-year college degree, 115 had a Master’s degree, and 31 had a PhD or professional degree.

In terms of the political leanings of the participants, 325 were registered Democrats, 160 registered Republicans, 136 Independent/Other, 112 unaffiliated, and 43 not registered. 430 voted for Biden, 205 for Trump, and 145 did not vote.

#### Design

Materials for this study can be found on OSF (https://osf.io/gq8f2/).

**Fig. 5:**
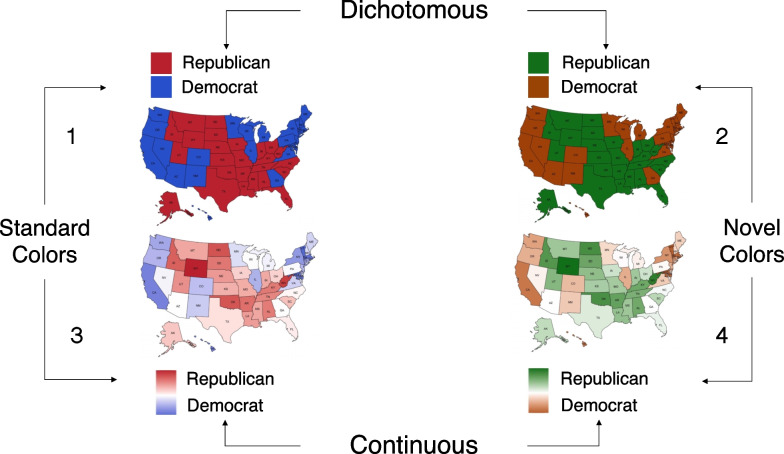
Sample Study 2 Task stimuli for each of the four between-subject conditions: (1) red/blue dichotomous, (2) red/blue continuous, (3) orange/green dichotomous, and (4) orange/green continuous. Within the four, hue varying conditions, the colors assigned to each state were randomized across participants, but the states were always divided evenly across the two hue categories. Participants completed 15 trials, which consisted of estimating fifteen randomly assigned States out of the total Fifty U.S. States. On each of the fifteen trials, the position of the line with the arrow identified which of the fifteen states the participant was judging. Note that the colors assigned to each state remained constant across a single participant’s trials based on the actual results of the 2020 Presidential election

The task used in Study 2, similarly to Study 1, involved a 2 × 2 between subjects design: Hue Pair (red/blue versus orange/green) × Gradient Steps (dichotomous versus continuous) (Fig. 5). Note that we removed the grey map. However, in this study, participants were instructed to use the maps to estimate the actual Democrat/Republican voting margins in the 2020 presidential election for 15 randomly-selected U.S. states (MIT Election Data & Science Lab, 2017). Unlike in Study 1, the maps in this study were accompanied by a legend specifying the correspondence between hue and political party, as depicted in Fig. 5. In the dichotomous conditions, the legend included only the two base colors, and in the continuous conditions, the legend contained a continuous gradation between each base color (through white as the center point). Each of the 50 states were labeled with their two-letter initials on the map.

On each trial participants were randomly assigned a particular state (e.g., Montana (MT)). They were first instructed to click on the state on the initialed map to make sure they saw its color. Then, they were asked to estimate what percent of votes went to Democratic vs Republican candidates (they were told to ignore third-party votes, so the major party votes added up to 100%). Participants were given the following instructions: “Using the scale below, estimate the percent of the specified state's population that voted for the Democratic candidate and the Republican candidate in the 2020 presidential election. Remember that this is a map of the 2020 presidential election with each color representing the winning party.” On each trial, we also asked participants: “Imagine you were a resident of that particular state. How influential would your vote have been in that election?” on a 5-point scale (1—Not at all Influential, 5—Extremely Influential).

Following completion of the Estimation Task, participants saw an attention check that asked them to click the state of California on a labeled map. Of the 809 participants who made it to this point, 798 passed the attention check. We further removed 18 participants who reported using outside help (i.e., google), as preregistered.

Finally, participants completed an “End of study questionnaire,” similar to the one described in Study 1.

#### Hypotheses

We aimed to replicate the main findings from the first study: (1) The effect of hue pair; whereby red/blue judgments were more polarized than orange/green judgments; (2) The interaction between Gradient Steps and Hue Pairs, demonstrating that the continuous gradient red/blue maps were significantly less polarizing than dichotomous red/blue, but that there was not a significant difference between continuous orange/green maps and dichotomous orange/green maps. We pre-registered an additional hypothesis about Gradient Steps influencing perceived voting influence. We predicted that using dichotomous gradient steps would decrease perceived voting influence compared to continuous gradients steps.

#### Calculating polarization

Unlike in Study 1, which asked about a hypothetical election, Study 2 asked about a past election so there is a ground truth for comparison. We first took the state-by-state 2020 election results, removed third-party votes, and calculated the percent of votes that went to Biden, the Democratic candidate (divided by total votes for Biden and Trump, the Republican candidate). We then subtracted these state-level values from participants’ estimates of the percent that voted Democratic. More positive values meant that participants overestimated Democrat votes and more negative values meant they underestimated Democrat votes. To calculate how much participants overestimated the margin regardless of the actual winner, we reverse-scored the states that Republicans won by multiplying by − 1. The resulting values were our polarization outcome variable. This variable, as the deviation from true 2020 election results, is different from the polarization variable in Study 1 which was the deviation from the midpoint (50%).

We also calculated a variable called “actual voting margin”, which tells us by how much the winner of each state actually won as a deviation from 50% (e.g., if Trump won a state 52% to 48%, the actual voting margin is 2%). This variable, which we used as a moderator in our analyses, represents how polarized each state actually was in 2020.

## Results

Using Hue Pair and Gradient Steps as our independent variables, we ran two linear mixed effects models to predict perceived voting polarization and perceived voting influence (Bates et al., 2015). We included random intercepts for participants and states and used Satterthwaite’s method for approximating degrees of freedom (Kuznetsova et al., 2015). We further tested whether actual voting margin difference moderated the effects of Hue Pair and Gradient Steps on perceived voting polarization and perceive voting influence.

### Perceived voting polarization

**Fig. 6:**
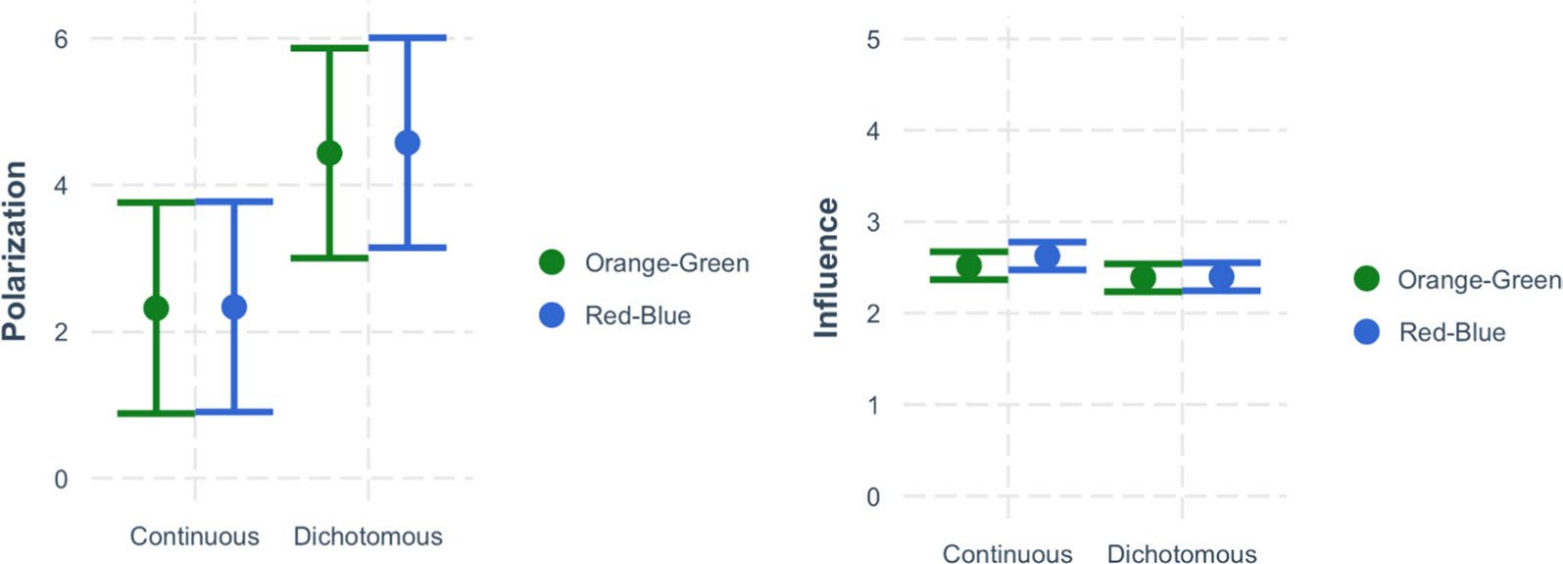
Study 2 results. The effect of Gradient Steps (continuous versus dichotomous) on the polarization of participants’ voting estimates (left) and how influential they thought individual votes would be (right). This effect was not moderated by whether the maps used a red/blue or orange/green hue pair

**Fig. 7:**
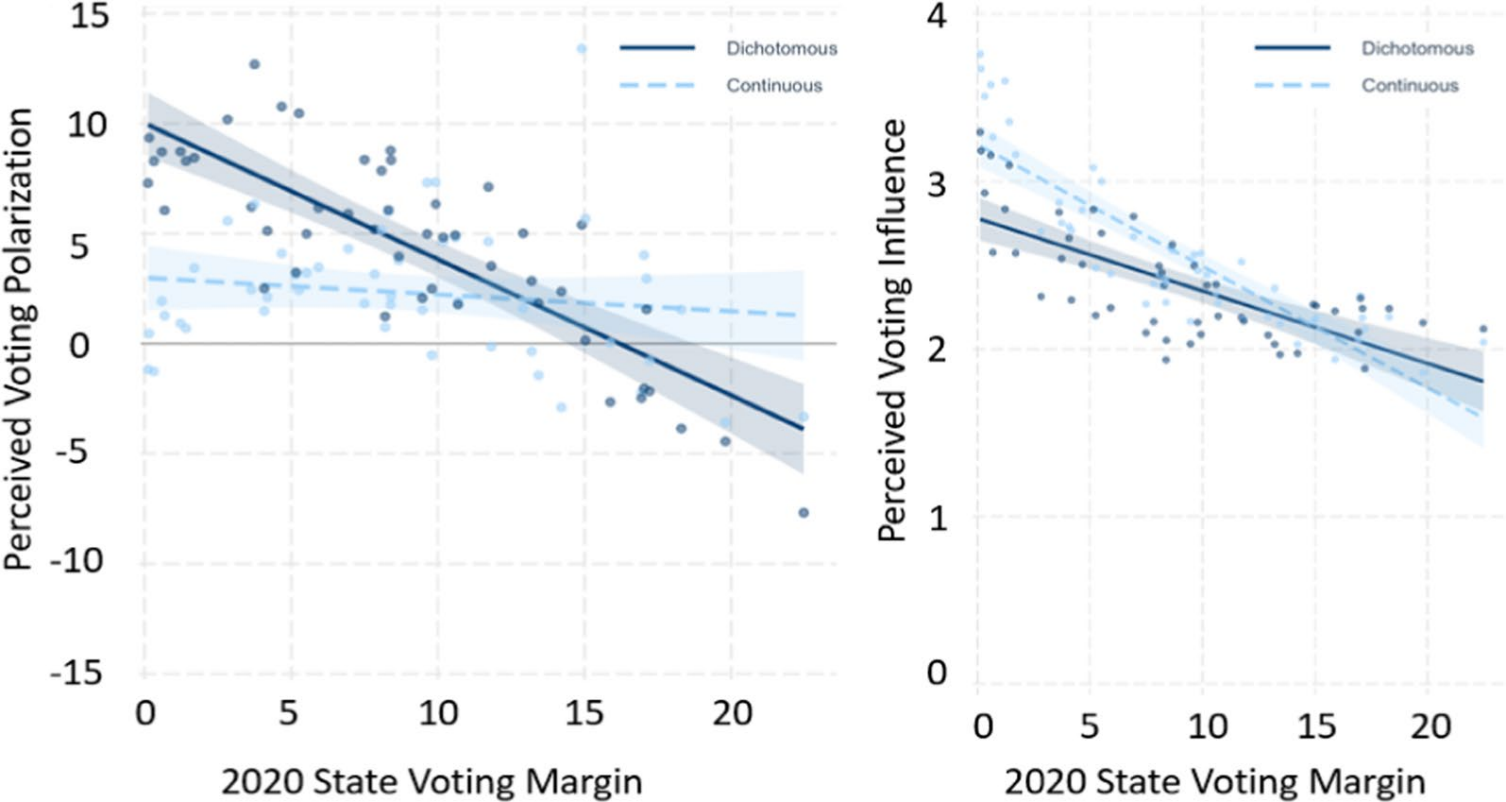
The Plots depict the interaction between Gradient Steps (continuous versus dichotomous) and the true polarization of U.S. states in the 2020 presidential election, in predicting participants’ estimates of how polarized the states were (on the left plot) and participants’ perceived voting influence for each state/trial (on the right plot). Each State is represented twice, once as a dark dot for the average dichotomous voting margin and once as a light dot for the average continuous voting margin. Overall, the left plot demonstrates that dichotomous maps exacerbated the polarization of participants’ estimates except for a few extremely polarized states for which dichotomous colors led participants to underestimate polarization. The plot on the right demonstrates that perceived voting influence (measured on a 5-point Likert Scale) increased when continuous gradient steps were used as opposed to dichotomous gradient steps, but the effect is particularly strong in the states  that had less (actual) voting polarization during the 2020 presidential election

As hypothesized, the effect of Gradient Steps significantly influenced perceived polarization, *b* = 2.18, *SE* = 0.591, *t*(775.72) = 3.680, *p* < 0.001. However, contrary to our hypothesis, there was no significant effect of Hue Pair, *b* = 0.08, *SE* = 0.591, *t*(775.53) = 0.133, *p* = 0.894, and no significant interaction between Hue Pair and Gradient Steps on perceived voting polarization, *b* = 0.12, *SE* = 1.183, *t*(775.50) = 0.104, *p* = 0.917 (see Fig. 6). Furthermore, when including actual voting margins as a moderator, we found a significant interaction between actual voting margin and Gradient Steps, *b* = − 0.579, *SE* = 0.039, *t*(10,990) = − 14.980, *p* < 0.001. This interaction indicated that dichotomous maps polarized estimates for 43 states, but for the 7 states with the highest voting margins, dichotomous maps led people to underestimate how polarized those states were (see Fig. 7).

### Perceived voting influence

As hypothesized, the effect of Gradient Steps significantly influenced perceived voting influence, *b* = − 1.79, *SE* = 0.05 *t*(776.23) = − 3.49, *p* < 0.001. There was no significant effect of Hue Pair, *b* = 0.06, *SE* = 0.52, *t*(776.08) = 1.121, *p* = 0.263, and no significant interaction between Hue Pair and Gradient Steps on perceived voting influence *b* = − 0.09, *SE* = 1.105, *t*(776.06) = − 0.904, *p* = 0.366 (see Fig. 6). Furthermore, when including actual voting margins as a moderator, we find a significant interaction of voting margin and gradient, *b* = 0.028, *SE* = 0.003, *t*(1.096e+04) = 9.499, *p* < 0.001. As with the Polarization dependent variable, continuous gradient maps led participants to believe that votes in a state matter more, unless the state’s voting margins were extremely high, at which point the continuous map simply emphasized for the participant how polarized the state truly was.

## Discussion

Study 2 increased the ecological validity and generalizability of our findings because participants estimated the actual voting margins of the 2020 presidential election across a randomized sample of all 50 states. We found that depicting electoral results using a continuous gradient significantly decreased perceived voting polarization as well as significantly increased perceived voting influence. We did not find an effect of novel hue pair (orange/green) versus the commonly used hue pair (red/blue) on either perceived polarization or voting influence. The conflicting findings between the significant effect of hue in Study 1 and the non-significant effect of hue in Study 2 suggests that, when participants are presented with a novel hue pair *and* a legend (as in Study 2), participants are quick to associate the categorical (dichotomous) party information with any hue scheme. Switching to a new hue scheme without strong semantic associations is therefore unlikely to solve issues of exaggerated perceived voting polarization.

## General discussion

Two decades ago, the United States media chose red to represent a majority of Republican votes and blue to represent a majority of Democratic votes, perhaps without realizing that the dichotomization of the country into states as red or blue would come to mislead the public into over-estimating voting polarization and perceive reduced voting influence. The goal of the present studies was to test the hypothesis that any overestimation of political polarization is in part due to the way the media has chosen to visualize election results on maps. Specifically, we asked whether people perceived voting margins to be more polarized because of their learned associations between blue-Democrat and red-Republican, and/or because red/blue maps dichotomize continuous variability in voting margins. Across two studies, we found that continuous color maps, compared to dichotomous color gradient maps, lead people to make less polarized voting estimates (hypothetical predictions in Study 1 and actual retrospective estimates in Study 2). In Study 2, we showed that dichotomous maps not only lead people to over-estimate how polarized US states were in the 2020 general election, but it also led them to think their votes would have mattered less.

The significant effect of hue pair (green/orange vs red/blue) that we found in Study 1 did not replicate in Study 2, in which participants were provided a legend that explicitly assigned a political party to each color. This inconsistency suggests that Study 1 participants either did not consistently assign the green/orange hues to Republican or Democrat party during the task, and/or did not pay attention to the hue information on the map. The null effect of Hue Pair in Study 2 suggests that the previously held semantic association between Republican-red and Democrat-blue does not exacerbate perceptions of political polarization when the assignment of parties to colors is explicitly labeled.

Instead, it appears that an information theory account of these findings would suggest that using continuous hue-lightness gradients simply provides more granular information, which in turn helps viewers of the map situate their voting estimates with greater accuracy compared to dichotomous maps. That is, when participants are presented with a dichotomous (red–blue) map and asked to estimate the voting margin for a particular state (e.g., a state represented in red), they can only infer that the voting margin will fall between 51 and 100% for republicans. In contrast, participants presented with a continuous map benefit from additional data granularity provided by the hue-lightness gradient steps (e.g., 50–62.5, 62.5–75, 75–87.5, 87.5–100).

The abovementioned explanation, would suggest that the observed polarization (i.e., overestimation of voting margin differences) is due to a lack of accuracy driven by the amount of information conveyed by continuous vs dichotomous maps, which would entail that in highly polarized states participants *underestimate* polarization (i.e., voting margin difference). However, results from study 2 demonstrate that participants in the dichotomous conditions *overestimated* the margins by which each state was won for almost all states (43 out of 50; see Fig. 7). The dichotomous maps could have simply narrowed the range of margins participants estimated without increasing them, but instead they biased participants to systematically overestimate between-state voting polarization.

An information theory account of this finding would further explain that without any prior information, participants in the dichotomous condition may have guessed the voting margin to be closer to the average estimate of 75.5% (i.e., the middle—between 51 and 100%) which would make their guess higher than most states’ actual voting margins. Furthermore, it is worth noting that the continuous hue-lightness gradient maps did not *eliminate* the overestimation of polarization, as most states were still judged, on average, to have higher voting margins in the 2020 election than they actually did. This could be due to the presence of *any* hue variation between states (as opposed to just white) being interpreted as stating a *greater* difference, which would slightly bias the voting margins upwards for the party associated with that particular hue.

An alternative explanation for the systematic over-estimation of voting margin polarization could be due to inflated perceptions of political polarization between Republicans and Democrats, which inadvertently leads participants to perceive voting estimates between red and blue states as more polarized than they are in actuality (see Rutchick et al., 2009 for initial findings supporting this hypothesis). Intractable conflict over partisan issues such as healthcare, gun control, and immigration plagues contemporary U.S. politics (Jacobson, 2016; Klein, 2020). This conflict is in part due to increased polarization between Democratic and Republican voters and legislators (Pew Research Center, 2014). However, people also overestimate the true degree of polarization (Westfall et al., 2015). It turns out that the majority of people hold more moderate views than others attribute to them (Blatz & Mercier, 2018; Fiorina & Abrams, 2008; Robinson et al., 1995). And expecting the rest of the population’s attitudes to be polarized, regardless of the true polarity of the population, leads to increased political action, such as attending political rallies and donating money to parties and candidates (Westfall et al., 2015). Polarized perceptions can also escalate conflict (Reeder et al., 2005; Robinson & Friedman, 1995). The concerning implications that stem from exaggerated public perceptions of polarization have increasingly attracted the attention of social scientists (Enders & Armaly, 2019; Lees & Cikara, 2021; Levendusky & Malhotra, 2016; Prior, 2013; Rutchick et al., 2009) and warrant further investigation in influencing perceived voting estimates.

In sum, an information theory account would suggest that participants’ voting estimates are simply inaccurate due to a lack of data granularity which also leads participants to guess a higher voting average difference, resulting in the polarization of voting margin estimates. In contrast, a polarization account would suggest that political polarization is made particularly salient through the dichotomization of political maps and therefore further polarizes voting margin estimates. Both accounts could be involved in explaining participants' systematic over-estimation of voting polarization, but future research is needed to further distinguish their individual or paired influence on voting estimates.

The present research also aimed to test a practical solution to reduce perceived voting polarization and increase perceived voting influence. Instead of using continuous purple maps as in prior work (Rutchick et al., 2009), which do not clearly communicate the winning majority party in a particular state, we used a gradient going from red to blue through white, which is more informative given the US state-by-state winner-takes-all election system. Across both studies, we found that using a continuous gradient instead of using dichotomous gradient steps can decrease perceived voting polarization (Study 1 and Study 2), along with increasing perceived voting influence (Study 2). Furthermore, the fact that hue association did not significantly influence perceived voting polarization and perceived voting influence when hue-party assignments were labeled in a legend suggests that political map designers do not need to stray from the commonly used hue schemes to reduce perceived voting polarization and influence.

In the present studies, we used color as the specified continuous visual feature, because color is typically used to represent election outcomes on maps. However, we suspect the present findings would generalize to any comparison of visual features that are dichotomized versus continuous. Future research could test this hypothesis by varying the type of visual features used to represent election outcomes (e.g., size, texture). The effects of dichotomization may also extend beyond visual features to other means of reporting election outcomes, such as numeric or verbal reports.

In a time where US. politics appear to be deeply divided; we conducted the present study to draw attention to the ways the usage of color in political maps might play a role in exacerbating polarization. Interestingly, we found that perceived voting polarization is partly driven by the information concealed in the dichotomous representation of continuous data, but is not influenced by the conditioned semantic associations between red/blue and Republican/Democrat political parties when the meanings of colors are labeled. The present results offer a simple way to reduce the polarizing effect of dichotomous color maps and increase perceived voting influence.

## Supplementary Information

**Additional file 1.** Supplemental Materials.

## Data Availability

All anonymized data and materials are available on OSF at the following link: https://osf.io/gq8f2/ All code is available on GitHub at the following link: https://github.com/adriennewood/red-state-blue-state/blob/master/README.md.

## Footnotes

1 Note that we asked participants to identify and select (click) the target state for every trial before making an estimate with the goal of ensuring that participants were looking at the map. Upon looking at the results for 10/50 states for each of the 4 conditions, we estimate that only 1.1% of the clicks seemed to widely deviate from the average target click. Given the small proportion of clicking errors as well as the practical constraints of precisely identifying each incorrect click for ≈ 14,000 trials, we included participants whose clicks did not align on the labeled target states.
