# Supplementary material for: Red and blue states: dichotomized maps mislead and reduce perceived voting influence
Source: Cogn Res Princ Implic. 2023 Feb 9;8:11. doi: 10.1186/s41235-023-00465-2 (PMC9908792; doi:10.1186/s41235-023-00465-2)
Supplement: Supplementary file 1 — Additional file 1. Supplemental Materials. [file 41235_2023_465_MOESM1_ESM.docx]

**Supplemental Materials**

**Red and blue states: dichotomized maps mislead and reduce perceived voting influence**

**Study 1**

Study 1 Distributions of Results by conditions _______________________________________________2

Calculating Polarization_________________________________________________________________4

Mean (Table S1.) and Standard Deviations (Table S2.) of Voting Predictions by condition_____________6

Base Rate Task________________________________________________________________________6

Exploratory analyses of the effect of color scales on polarization________________________________8

**References**

References___________________________________________________________________________6

**Study 1**

**Study 1 Distributions of Results by conditions**

The figure below (S.1) demonstrates the distributions of predicted voting percentages for the 5 conditions. Under each distribution, the dots represent (on average) the predicted voting % for each state (initialed) depending on being assigned as one of the two colors (blue/red) or (orange/green). For example, one can observe under the dichotomous red blue distribution that North Carolina (represented as NC) has an average democrat voting percentage around 54% when represented in blue, but 45% when represented in red.

**Figure S1. Distributions of average individual state voting predictions by conditions (Study 1)**


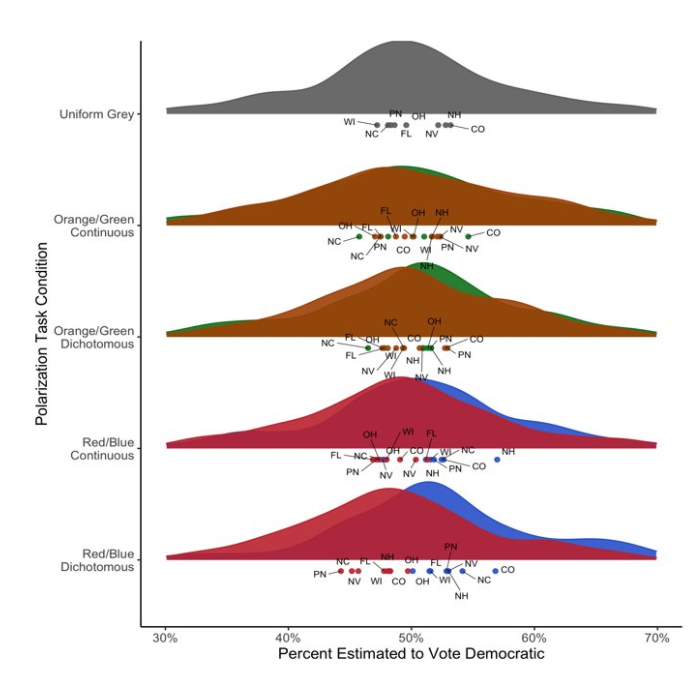


**Calculating Polarization**

Participants were randomly assigned to one of four conditions: dichotomous red-blue dichotomous orange-green, continuous red-blue, continuous orange-green, and uniform grey. Each participant completed 8 trials, judging one state on each trial. We calculated how “polarized” a participant’s voting predictions were by calculating the relative variability in their responses for all the states depicted with the same hue versus the same states randomly depicted with the alternative hue. In other words, we conducted an ANOVA separately for each participant (Ntrials=8), and used the resulting F statistic, which indicated how distinct their judgments were for each map color, as our measure of polarization (see Figure S2 for a visual explanation).

We calculated secondary polarization scores with the aim of distinguishing between two different types of potential polarization: spread and clustering. The F statistic, our primary measure of polarization derived from an ANOVA, is calculated by dividing the Mean Squares Between (MSB) by the Mean Squares Within (MSW). MSB is calculated by dividing the Sum of Squares Between (SSB) by the Degrees of Freedom Between, while MSW is calculated by dividing the Sum of Squares Within (SSW) by the Degrees of Freedom Within. Polarization, as measured by calculating an F statistic for each participant based on their responses across the 8 trails, can be influenced by both spread (measured by SSB) and clustering (measured by SSW), which are two different features of polarization. The SSB for a participant captures the average variation of their voting margin predictions between states that are randomly assigned to different colors. The SSW for a participant captures the average variation for which a participant predicts states of the same color to vote.


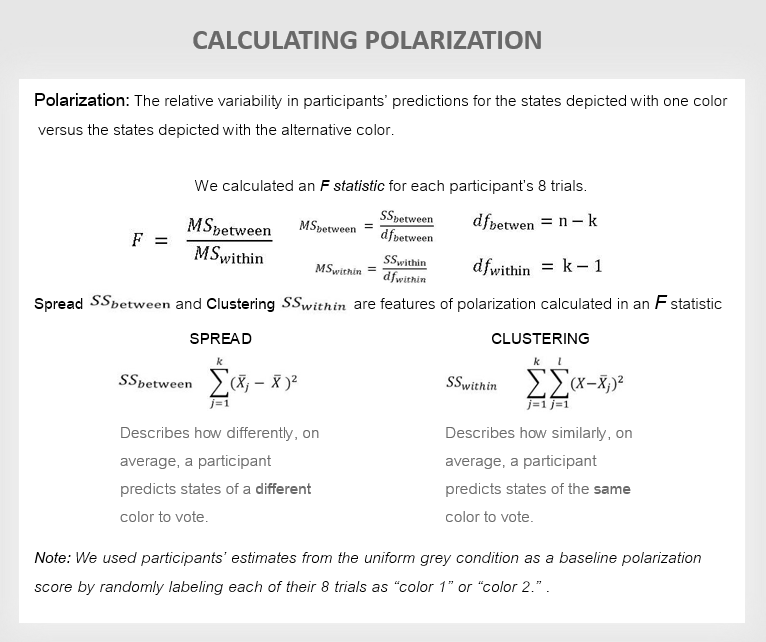


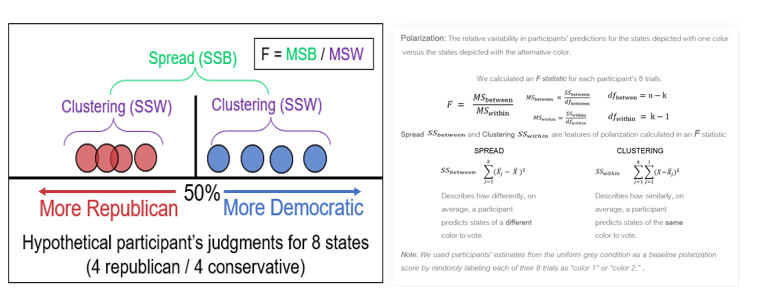


**(4 Republican / 4 Democrat)**

**Figures S2.** Each trial (N=8) for a participant in the dichotomous Red-Blue condition is depicted on a voting margin continuum above. 4 states are depicted as red circles and 4 states are depicted as blue circles. Clustering (purple) - measured with SSW - represents how close states of the same color were predicted to vote. Spread (green) - measured by SSB - represents how far apart states of different colors were predicted to vote.

**Table S1.** Study 1: Mean (Democrat) voting predictions by color and condition.


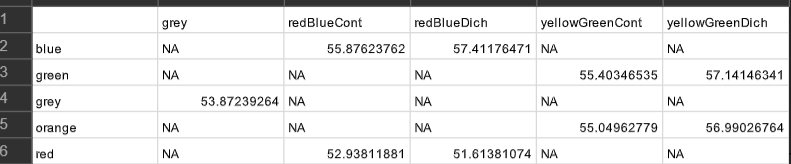


**Table S2.** Study 1: Standard Deviations around the mean (Democrat) voting predictions by color (hue) and condition.


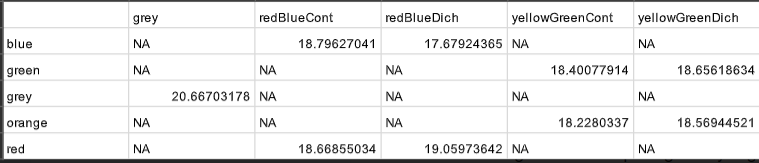


Note: “yellowGreen” (in tables S1 and S2) actually refers to the “orange-green” condition.

**Base Rate Task**

**Introduction.** The present study also examined a second potential cognitive consequence of red-blue maps: the salience of information conveyed via color might cause people to underappreciate the value of accompanying numeric information in predicting electoral outcomes. We asked whether dichotomous color maps might lead participants to ignore numerically reported base rates. For instance, participants may ignore information that 51% of a county voted Republican, and guess instead, that a random voter in the county has a substantially higher likelihood of being Republican, simply because the district is colored entirely in red. We hypothesized that base rate Neglect effects would be stronger for standard color scales (red/blue) than for alternative (novel) color scales (orange/green or uniform grey). Neglect effects would be stronger for standard color scales (red/blue) than for alternative color scales (orange/green or uniform grey).

**Base Rate Task Design.** Participants were told the percent of registered voters who voted Republican and Democrat in an unnamed U.S. county and then estimated the likelihood of a generic person voting Republican or Democratic. The target’s views were represented in a vignette placed below a single state outline which has voting percentages depicted on it (see **Figure S3**). This task consisted of
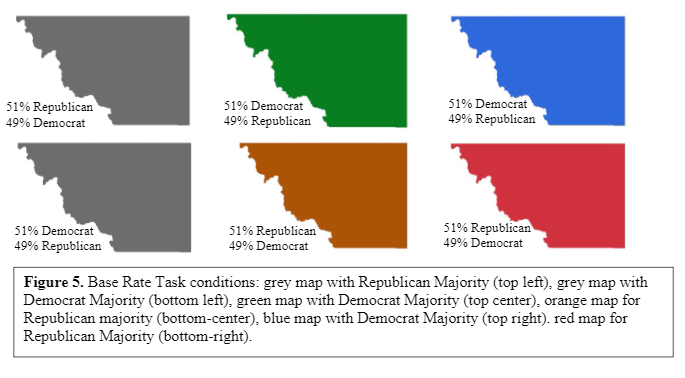
a single trial.

**Figure S3.**

***Map stimuli.*** We used a map of Loving County TX (see **Figure S3**) in order to minimize participants’ prior knowledge compared to using any given state (the county shape remained nameless when presented to participants). Participants were not told the name or location of the county. There were 6 between-subject conditions. Participants’ color condition assignment from the eight states in the Polarization Task was the same as the color condition for the single county presented in the Base Rate Task. For example, participants who saw the orange/green colors during the Polarization Task were randomly assigned to a green or orange county for the Base Rate Task. The red-blue and orange-green maps were generated by filling the county with colors: red, blue, orange, green and grey (same values as in the Polarization Task).

***Procedure.*** Before the Base Rate Task began, participants were told, “You will now see a map of a U.S. County. The numbers reported below the map indicate the percentage of the state's registered voters (excluding Independents) who are Democrats or Republicans.” Below the map was a description of Chris, a hypothetical resident voter of the county. The description outlined Chris’s views on 5 relatively non-partisan political issues: “the government should implement new legislation to protect domestic animals”, “The government should increase spending on support for veterans”, “The government should update the aging infrastructure”, “the government should simplify the tax system, allowing easier tax filing”, “the government should focus on strengthening the economy” (Pew RESEARCH Center, 2020).

Participants then estimated the resident’s likelihood of being a registered Democrat or Republican on two slider scales ranging from 0% (“Likelihood of being Democrat”) to 100% (“Likelihood of being a Republican). Subsequently, participants were asked to answer, “How influential will Chris’ vote be in the upcoming county wide election?” on a slider scale ranging from 0% (“no influence”) to 100% (“a great deal of influence”). This question was meant to capture the downstream effects of predicted polarization on perceived voting influence. If states are predicted to be more polarized (i.e., having a larger margin of difference) each individual vote may be perceived as less influential.

**Base Rate Task preregistered analyses.** The Base Rate Task tested the prediction that, compared to neutral and orange or green color-coded maps, red or blue color-coded maps cause participants to ignore base rate statistics to a greater degree.

The six possible maps (red R majority, blue D majority, orange R majority, green D majority, grey R majority, grey D majority) can be described as the interaction between two variables: Majority Condition (Democrat majority = -.5 and Republican majority = .5) and Color Condition (a dummy-coded variable with levels for red/blue, orange/green, and uniformly grey, with uniformly grey as the reference level). Because the uniformly grey condition is the reference level for the Color Condition, the main effect of Majority serves as an attention/manipulation check on whether people paid attention to the reported base rates for the hypothetical county. We assumed that participants exposed to a uniformly grey county with a Republican majority would rate the voter as likelier to vote Republican than participants exposed to a grey county with a Democratic majority. The interaction between Majority Condition and the uniformly grey versus red/blue dummy variable tested hypothesis 4, that participants would overestimate the voting margin (ignoring the provided base rate percentage for the county) when the map was colored as red or blue, compared to when it was uniformly grey.

**Results.** The base rate manipulation failed: for the grey maps, participants’ judgments of Chris’s political leaning were not significantly influenced by whether they were told Chris’s county was 51% Democrat or 51% Republican, *b* = 1.480, *SE* = 4.588, *t*(494) = 0.32, *p* = .747. Thus, although the interaction between Color Condition and Majority was not significant, *b* = 0.096, *SE* = 5.647, *t*(494) = 0.017, *p* = .987, we cannot draw any conclusions about Hypothesis 4. The dummy variable comparing grey and red/blue maps was significant, *b* = -6.114, *SE* = 2.823, *t*(494) = -2.17, *p* = .031, η_p_^2^ = .011. Controlling for whether Republicans or Democrats were represented as the numeric majority, Chris was judged as likelier to be Republican when the map was grey (*M* = 60.84%, *SD* = 21.44%) compared to red or blue (*M* = 54.74%, *SD* = 23.81).

Finally, we ran an identical model as before with participants’ estimates of how influential Chris’s vote would be as the outcome variable. None of the main effects or interaction terms were significant, *p*’s >= .404.

**Discussion.** The second task was intended to test whether party associated red-blue maps would cause participants to ignore base rate statistics more than orange-green or grey maps. The results are inconclusive because we did not successfully manipulate base rates in the control (i.e., neutral map) condition. We further note that the hypothetical voter was on average perceived as being more Republican across conditions. Future studies could improve on the experimental design by sampling many opinions and pre-testing them to be sure that they are non-partisan.

**Exploratory analyses of the effect of color scales on polarization**

The following analyses explored possible moderators of the effects of the Polarization Task experimental manipulations.

*Does being a Trump vs. Biden supporter moderate responses?* We reran Model 2 with an additional variable coding whether the participant supported Biden (-0.5) or Trump (0.5), which interacted with the original Hue Pair X Gradient Steps interaction. The model thus included a three-way interaction, all two-way interactions, and the three main effects. The interaction from the preregistered analysis still held, *p* = .038, as did the effect of Color Scale, *p* < .001. A notable, but marginally significant effect is found in the interaction between support for Biden vs. Trump and the Gradient Steps variable. This effect is slightly above the *p* < .05 threshold for significance, *b* = 0.262, *SE* = 0.125, *t*(348) = 1.89, *p* = .0599, η_p_^2^ = .010. Unpacking this marginally significant interaction term reveals Trump supporters’ predictions were more polarized by the continuous gradient (untransformed F-values: *M* = 2.04, *SD* = 3.84) versus dichotomous hue pairs (*M* = 15.64, *SD* = 69.82) manipulation than were Biden supporters’ (*M* = 4.49, *SD* = 16.55 and *M* = 4.70, *SD* = 25.19, respectively).

*Does concern about polarization predict the polarization of participants’ judgments?* We repeated Model 2 and added the four self-report questions about polarization concerns as covariates (“Do you think the United States is more politically divided now than in the past?”; “ Do you think political polarization is a problem in our country?”; “Do you think half of the country is being ignored by politicians?”; and “ Do you think Red state Americans and Blue state Americans ultimately share the same values?”). None of these measures of self-reported concern about polarization predicted participants’ actual judgment polarization, *p*’s >= .305.

**References**

“Political Polarization in the American Public.” Pew Research Center, Washington, D.C. (2014).

<https://www.people-press.org/2014/06/12/political-polarization-in-the-american-public/>
